# Supplementary material for: Antimicrobial and Amyloidogenic Activity of Peptides Synthesized on the Basis of the Ribosomal S1 Protein from Thermus Thermophilus
Source: Int J Mol Sci. 2020 Sep 2;21(17):6382. doi: 10.3390/ijms21176382 (PMC7504387; doi:10.3390/ijms21176382)
Supplement: Supplementary file 1 [file ijms-21-06382-s001.pdf]

Supplementary Table 1. Aggergation kinetics of the elongate peptides based on the ribosomal S1 protein of *T.thermophilus.*

1	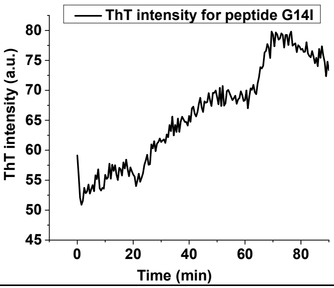	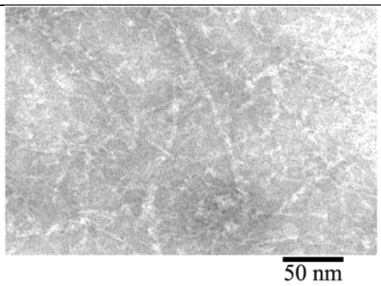	According to fluorescence spectroscopy data: the fluorescence intensity of thioflavin T (ThT) increases from 50 to 80 au. This indicates the presence of amyloids in the peptide preparation GlyGlySarGlyVTDFGVFVEI (G14I).<br>According to electron microscopy data: the (G14I) peptide GlyGlySarGlyVTDFGVFVEI make many thin prefibrills/fibrils, amorphous aggregates of peptides of different sizes.
2	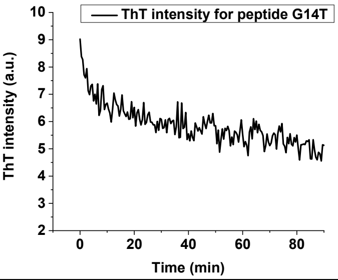	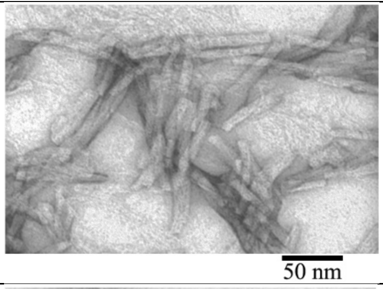	According to fluorescence spectroscopy data: the initial fluorescence intensity of ThT, which decreased from 9 to 4 a.u. indicate the presence of amyloids (which tend to disaggregate) in the peptide preparation GlyGlySarGlyVVEGTVVEVT (G14T).<br>According to electron microscopy data: the (G14T) peptide GlyGlySarGlyVVEGTVVEVT make short dense fibrils.
3	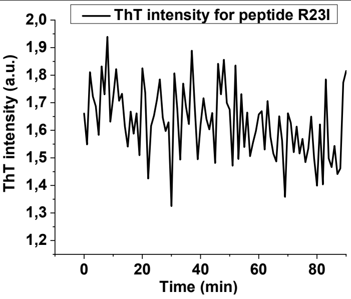	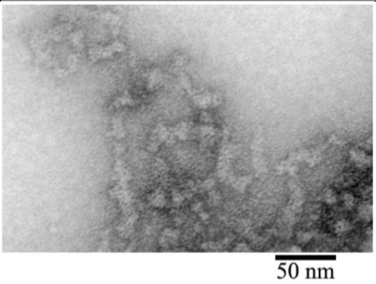<br>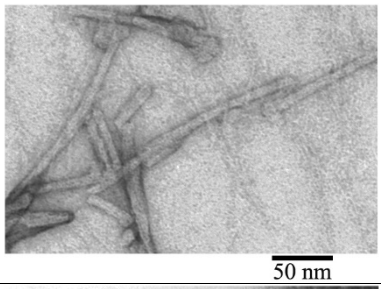	According to fluorescence spectroscopy data: the initial low intensity of ThT fluorescence is about 2 a.u. does not change significantly, which indicates the absence of R23I amyloids.<br><br>According to electron microscopy data: aggregates of different sizes and complexes of aggregates are present. Aggregates and fibrils may form depending on conditions.
4	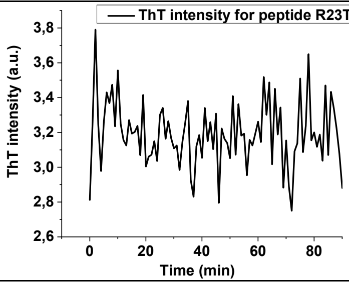	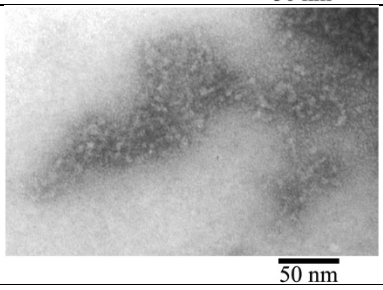	According to fluorescence spectroscopy data: the initial low intensity of ThT fluorescence is about 2 a.u. does not change significantly, which indicates the absence of R23T amyloids.<br><br>According to electron microscopy data: aggregates of different sizes and complexes of aggregates are present. There are no fibrils.

Supplementary Table 2. Experimental results of studying the kinetics of coaggregation of samples of amyloidogenic peptides based on the ribosomal S1 protein and the whole S1 protein of *T. thermophilus*.

<table>
<tr>
<td rowspan="4">1</td>
<td rowspan="4">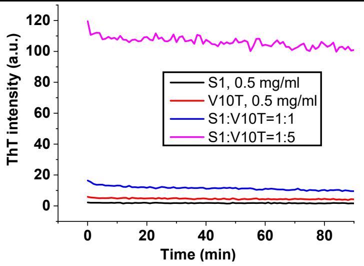
</td>
<td>S1 (0.5 mg/ml)
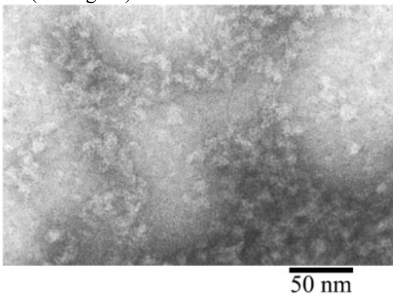
</td>
<td rowspan="2">According to fluorescence spectroscopy data: upon joint incubation of the S1 protein and V10T peptide, the fluorescence intensity of Thioflavin T (ThT) increases from 6 to 6 in a 1:1 ratio and from 6 to 120 a.u. in a ratio of 1:5. This indicates the presence of amyloids, which tend to disaggregate.

According to electron microscopy data:
The V10T peptide and the S1 protein form aggregates. Thus, it is noticeable that these aggregates have morphological differences (the components of the aggregates of the V10T peptide are slightly smaller than those of the S1 protein).</td>
</tr>
<tr>
<td>V10T (0.5 mg/ml)
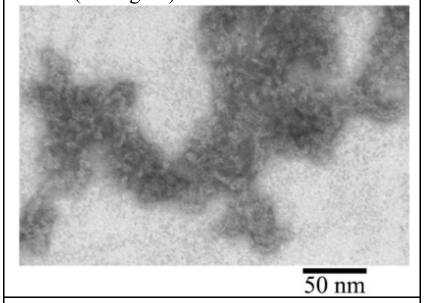
</td>
</tr>
<tr>
<td>S1:V10T=1:1 (0.5 mg/ml)
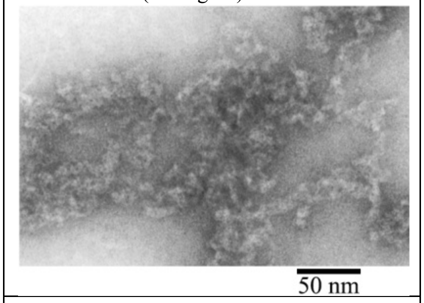
</td>
<td>In the mixture of the V10T peptide and S1 protein in a 1:1 ratio, only aggregates are present.</td>
</tr>
<tr>
<td>S1:V10T=1:5 (0.5 mg/ml and 2.5 mg/ml)
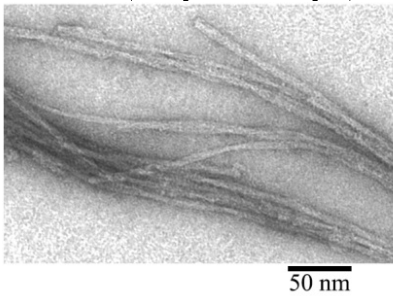
</td>
<td>In the mixture of the V10T peptide and S1 protein in a ratio of 5:1, both aggregates and fibrils are present.</td>
</tr>
<tr>
<td>2</td>
<td></td>
<td>S1 (0.5 mg/ml)
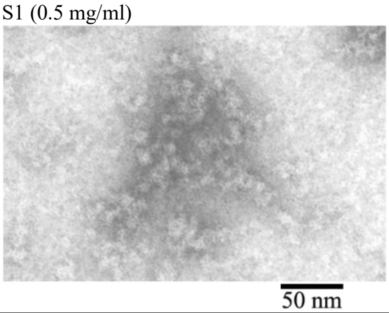
</td>
<td>According to fluorescence spectroscopy data: upon joint incubation of the S1 protein and G14T peptide, the fluorescence intensity of Thioflavin T (ThT) decreases slightly from 20 to 16 a.u. in a 1:1 ratio. This indicates the presence of amyloids, which tend to disaggregate.</td>
</tr>
</table>

	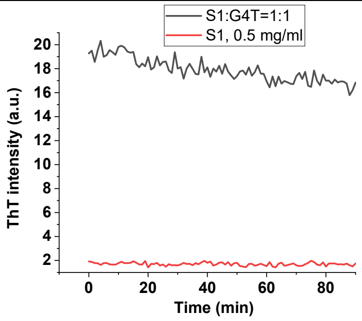	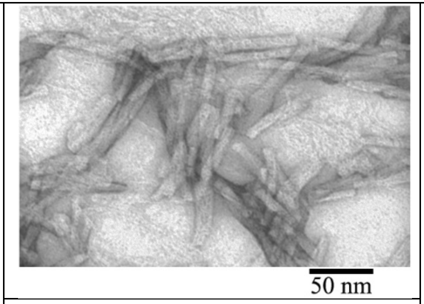<br>S1:G14T=1:1 (0.5 mg/ml and 5 mg/ml)<br>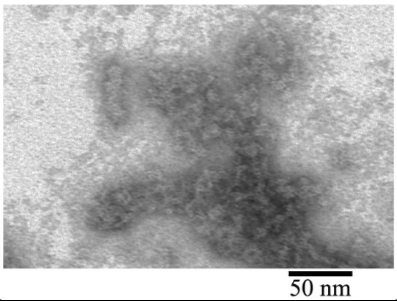	According to EM data, when the ratio S1:G14T = 1:1, fibrils are not formed and the morphology of aggregates is similar to the morphology of S1 protein.
4	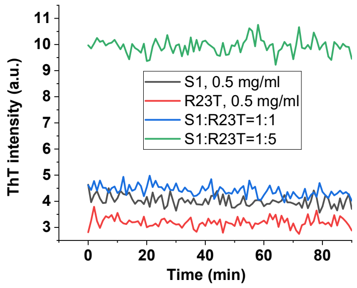	S1 (0.5 mg/ml)<br>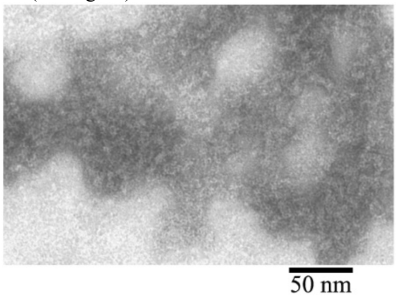	According to the data of fluorescence spectroscopy: upon joint incubation of The S1 protein and R23T peptide, the fluorescence intensity of Thioflavin T (ThT) increases insignificantly from 3-4 to 5 a.u. in a ratio of 1: 1 and up to 11 at a ratio of 1: 5, which indicates the absence of the formation of amyloids or their insignificant amount at a ratio of S1: 23T = 1:5.
		R23T (0.5 mg/ml)<br>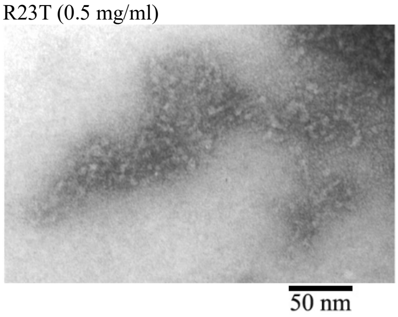	When the ratio S1:R23T = 1:1, aggregates are observed.
		S1:R23T=1:1 (0.5 mg/ml)<br>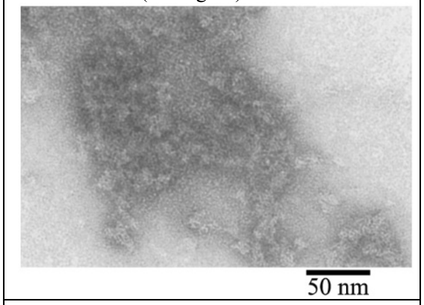	When the ratio S1:R23T = 1:5, aggregates of different morphology (type 1 and type 2), fibrils and thin films are formed.
		S1:R23T=1:5 (0.5 mg/ml and 2.5 mg/ml)<br><br>Aggregates of type 1	

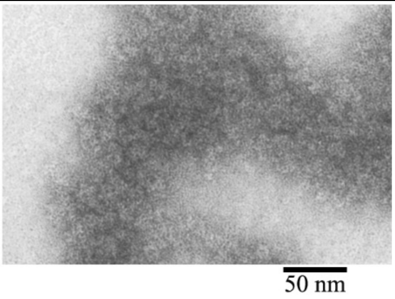

Aggregates of type 2

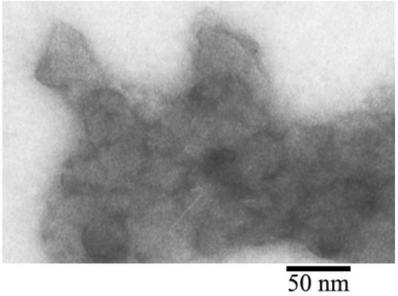

Fibrils

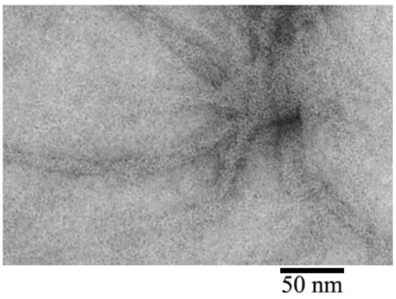

Films

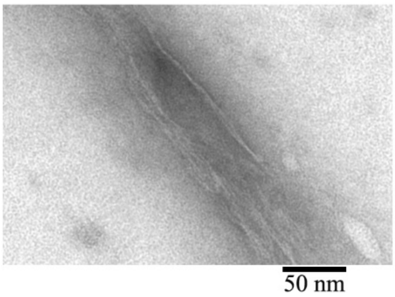

Supplementary **Table 3.** Statistical parameters of response curves.

Model	logLik	AIC	Res var
Kanamycin			
Log- logistic model with three parameters	16.94	-25.88	0.01
Exponential model with two parameters	10.46	-14.93	0.01
Model Michaelis-Menten with two parameters	7.91	-9.82	0.02
R23I			
Log- logistic model with two parameters	15.95	-25.90	0.02
Exponential model with two parameters	15.74	-25.49	0.02
Model Michaelis-Menten with two parameters	14.71	-23.42	0.02
R23T			
Log- logistic model with two parameters	4.70	-3.40	0.02
Exponential model with two parameters	4.35	-2.69	0.03
Model Michaelis-Menten with two parameters	3.68	-1.35	0.03

Supplementary **Table 4.** Number of proteins in different experimental groups.

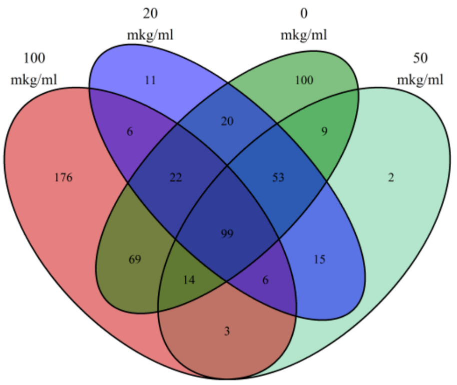

Concentration R23I	Total protein detected	Unique proteins	Common proteins for cells in control and treated cells
0 μg/ml	386	100	-
20 μg/ml	232	11	20
50 μg/ml	201	2	9
100 μg/ml	395	176	69

Supplementary **Table 5.** The distribution of proteins sensitive to the action of the R23I peptide.

Group	Keywords	Number of proteins			
		0 μg/ml	20 μg/ml	50 μg/ml	100 μg/ml
Biosynthetic processes, protein biosynthesis	Histidine biosynthesis	1	0	0	1
	Ribosome biogenesis	2	0	0	0
	Methionine biosynthesis	5	1	1	3
	Isoleucine biosynthesis	2	0	0	1

	S-adenosyl-L-methionine	1	0	0	1
	Threonine biosynthesis	4	1	1	2
	Aminoacyl-tRNA synthetase	20	7	7	19
	tRNA processing	1	0	1	1
	Aromatic amino acid biosynthesis	1	0	0	1
Processes associated with the conversion and metabolism of nucleic acids	Nuclease	1	0	0	2
	DNA replication	1	0	0	2
	Endonuclease	1	0	0	2
	Purine biosynthesis	3	1	1	3
	Purine salvage	1	0	0	0
	One-carbon metabolism	1	0	0	1
Processes associated with the conversion of polypeptide chains	Aminopeptidase	3	0	0	0
	Aminotransferase	1	0	0	1
	Protease	16	5	5	11
	Serine protease	7	1	2	3
	Hydrolase	26	9	8	31
The processes associated with energy metabolism, and factors determining enzyme activity	NADP	8	2	2	6
	Glyoxylate bypass	4	1	1	4
	Phosphoprotein	3	1	2	5
	Potassium	1	0	0	1
	Redox-active center	3	0	0	3
	Serine/threonine-protein kinase	1	0	0	0
	Magnesium	23	9	9	33
	Allosteric enzyme	6	1	1	4
